# Use of thromboelastography before the administration of hemostatic agents to safely taper recombinant activated factor VII in acquired hemophilia A: a report of three cases

**DOI:** 10.1186/s12959-022-00387-x

**Published:** 2022-05-16

**Authors:** Hiroki Hosoi, Yuina Akagi, Toshiki Mushino, Masahiro Takeyama, Naoto Minoura, Takayuki Hiroi, Yoshiaki Furuya, Masaya Morimoto, Shogo Murata, Shinobu Tamura, Takashi Sonoki

**Affiliations:** 1 Department of Hematology/Oncology, Wakayama Medical University, Wakayama, Japan; 2 Department of Internal Medicine, Kainan Municipal Medical Center, Wakayama, Japan; 3 Department of Hematology, Kinan Hospital, Wakayama, Japan; 4 Department of Pediatrics, Nara Medical University, Nara, Japan; 5 Department of Clinical Laboratory, Wakayama Medical University Hospital, Wakayama, Japan

**Keywords:** Acquired hemophilia A, Thromboelastography, Recombinant activated factor VII

## Abstract

**Background:**

Acquired hemophilia A (AHA) is a rare autoimmune disease characterized by bleeding events. Recombinant activated factor VII (rFVIIa) is a first-line bypassing agent, which is effective against clinically significant bleeding. However, there is no standard way of tapering and discontinuing rFVIIa, mainly because there is no established method for monitoring rFVIIa therapy for AHA.

**Case presentation:**

Here, we report three AHA cases, in which we adjusted the rFVIIa dosing interval based on the results of thromboelastography (TEG) performed just before the administration of the next dose of rFVIIa. The dosing interval of rFVIIa was prolonged based on the reaction rate time (R) according to TEG, which is correlated with coagulation factor activity. The R-value reference range reported by the manufacturer of the TEG system was used as a threshold for making decisions. In these three cases, there was no rebleeding, and the patients’ ability to perform activities of daily living did not decline.

**Conclusion:**

Our cases suggest that conducting TEG-based monitoring just before the administration of the next dose of rFVIIa may be useful for guiding increases in the rFVIIa dosing interval without causing rebleeding events. Further investigations are warranted to examine how TEG could be used to determine the most appropriate rFVIIa dosing interval, e.g., through regular TEG-based monitoring, and the optimal TEG-derived threshold for indicating changes to the rFVIIa dosing interval.

## Background

Acquired hemophilia A (AHA) is a rare disease characterized by severe bleeding and a high risk of mortality. It is an autoimmune disease caused by the spontaneous production of neutralizing inhibitors against endogenous factor VIII [1]. In addition, it is commonly associated with autoimmune disorders, solid tumors, lymphoproliferative disease, and pregnancy; however, approximately 50% of cases develop in the absence of any apparent underlying conditions [2, 3]. Severe bleeding events can lead to a decreased ability to perform activities of daily living (ADL). Thus, it is crucial to rapidly control bleeding and prevent recurrent bleeding. Until coagulation inhibitors have been eradicated by immunosuppressive therapy, hemostatic therapy should be administered appropriately to control bleeding [4]. Recombinant activated factor VII (rFVIIa) is used as a bypassing agent during hemostatic therapy in AHA patients [3, 5]. Although a higher single rFVIIa dose of 270 μg/kg was reported to be effective to treat bleeding episodes in congenital hemophilia with inhibitors, an rFVIIa dose of 90 μg/kg is considered to be the standard dose for AHA [6–8]. The recommended dose and dosing interval of rFVIIa are determined at the start of treatment [4]. However, there is no standard way of tapering and discontinuing rFVIIa, mainly because there is no established method for monitoring rFVIIa therapy for AHA. Thromboelastography (TEG) measures the clot-formation capacity of whole blood [9]. Although the use of TEG to assess the hemostatic effects of rFVIIa after its administration has been reported previously, there have been no reports about the utility of TEG for tapering and discontinuing rFVIIa [10, 11]. The half-life of rFVIIa is short. Therefore, we hypothesized that performing TEG just before the administration of the next dose of rFVIIa, rather than after the administration of rFVIIa, would facilitate safe increases in the rFVIIa dosing interval during the tapering of rFVIIa.

TEG is used to evaluate the global clotting function of whole blood. The TEG 6 s® system (HAEMONETICS, Boston, MA, USA), a relatively new TEG platform, is easy to use in clinical laboratories [12]. In this study, TEG was performed using kaolin and the TEG 6 s® system just before the administration of rFVIIa at our laboratory. Clotting time was evaluated based on the reaction rate time (R) according to TEG [9]. The R-value represents the time required to reach a clot amplitude of 2 mm. The R-value is defined as the time from the addition of calcium to the start of clot formation, which is correlated with coagulation factor activity [9, 13]. Here, we report three cases, in which we adjusted the rFVIIa dosing interval based on the results of TEG performed just before the administration of the next dose of rFVIIa.

## Case presentation

### Case 1

**Fig. 1:**
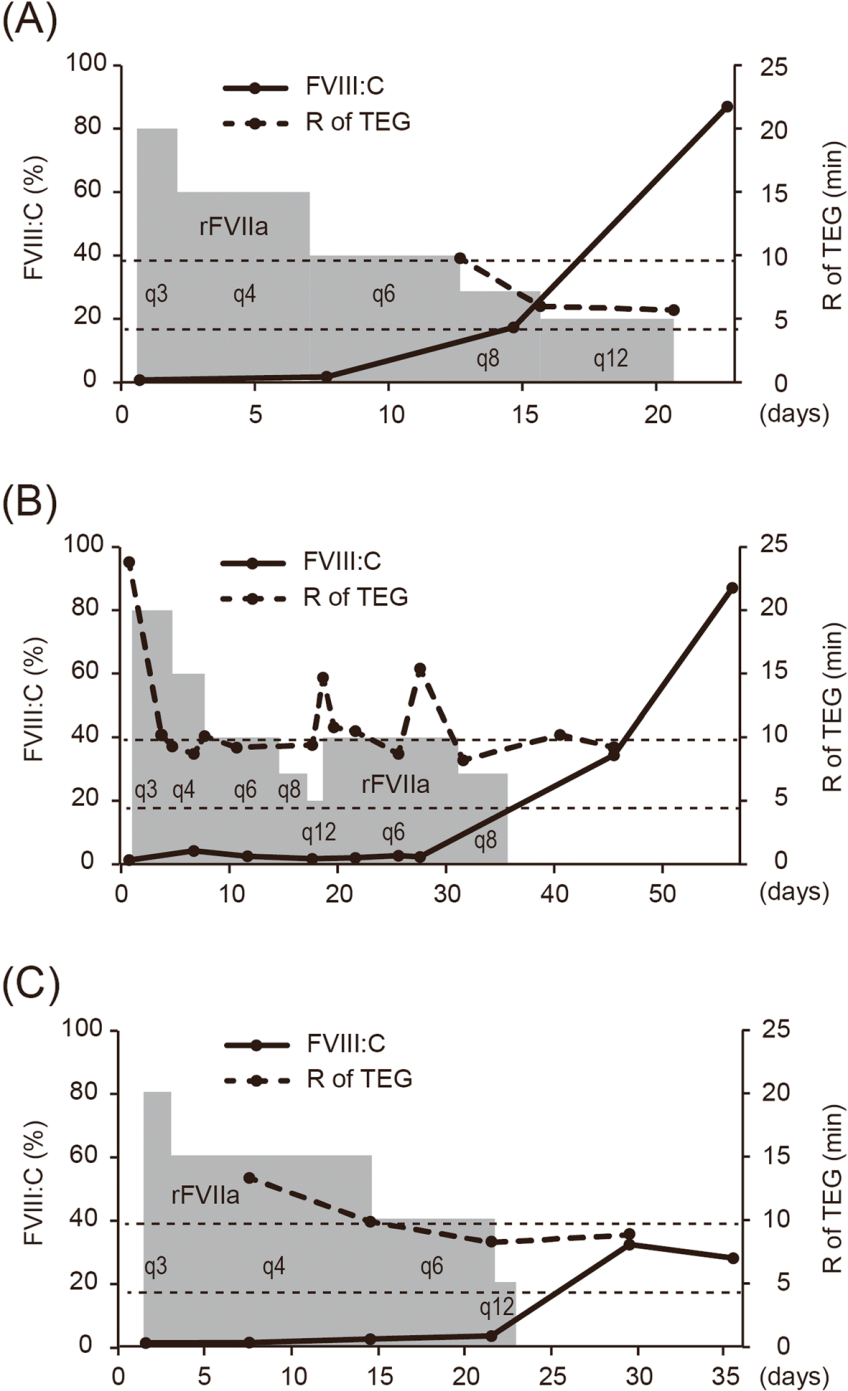
Change in the reaction rate time (R) according to thromboelastography (TEG)**.** The levels of factor VIII coagulant activity (FVIII:C) and the R-value according to TEG in three acquired hemophilia A patients (**A-C**) who were being treated with recombinant activated factor VII (rFVIIa) are shown. The doses of rFVIIa are also represented. The black line and dashed line represent dynamic changes in FVIII:C levels and the R-value, respectively. The x-axis indicates the number of days from the start of rFVIIa treatment until the FVIII:C level rose to > 20%. The day on which rFVIIa treatment was initiated was designated “day 1”. The horizontal dotted line indicates the R-value reference range (4.6-9.1 min) according to TEG. The administration of rFVIIa is indicated by grey boxes. “q3” means that rFVIIa was administered every 3 hours

An 82-year-old female was admitted for bullae with bleeding and a nasal hemorrhage. She had no relevant medical history other than a herpes zoster infection 3 years before admission. She was not taking any medication. She had developed bullae with bleeding 2 weeks before admission and saw a dermatologist. Laboratory tests revealed a prolonged activated partial thromboplastin time (APTT), and she visited our clinic. Coagulation tests showed a prolonged APTT (80 sec), reduced factor VIII coagulant activity (FVIII:C) (0.7%), and a high factor VIII (FVIII) inhibitor titer (5.4 Bethesda units [BU]/mL) (Table 1). She was diagnosed with AHA with bullous pemphigoid. Computed tomography (CT) revealed a local alveolar hemorrhage. rFVIIa (90 μg/kg) treatment was started, and the initial dosing interval was 3 hours. Immunosuppressive therapy with 1 mg/kg of prednisolone was also initiated. Before the administration of the first dose of rFVIIa, her clotting time according to rotational thromboelastometry (ROTEM) was extended (70.5 minutes). ROTEM, which was performed at a specialized laboratory, was only used to analyze the patient’s clotting time before the administration of the first dose of rFVIIa. After that, TEG-based analyses were carried out at our laboratory. On the second day of rFVIIa treatment, the subcutaneous bleeding improved, and the rFVIIa dosing interval was gradually increased. Once the dosing interval had reached 6 hours, it was extended based on the patient’s coagulation capacity according to TEG performed immediately before the administration of rFVIIa (Fig. 1A). Thirteen days after the start of rFVIIa treatment, the patient’s R-value was 10 min; i.e., it had almost returned to the reference range (reference range: 4.6-9.1 min). Therefore, the rFVIIa dosing interval was increased from every 6 hours to every 8 hours. At 21 days after the start of the rFVIIa treatment, the patient’s R-value before the administration of rFVIIa was found to be 5.4 min, and hence, rFVIIa was discontinued. The patient did not suffer from any alveolar rebleeding. After the discontinuation of rFVIIa, the patient’s FVIII:C level recovered (87%), and the FVIII inhibitor became undetectable (Fig. 1A). The patient’s ability to perform ADL did not decline, and she was able to return to her daily life quickly. The administration of corticosteroids improved both her AHA and bullous pemphigoid. 1 year after the onset of AHA, the steroid therapy was discontinued. 1 month later, her bullous pemphigoid worsened, but her AHA did not recur. Steroid therapy was resumed, and the recurrent pemphigus was ameliorated. After more than 2.5 years of treatment with maintenance doses of steroid therapy (7.5 mg of prednisolone), the AHA has not relapsed.

**Table 1:** Characteristics of 3 patients with acquired hemophilia A that underwent TEG-based analyses

Case number	Age	Sex	Symptoms	Coagulation tests performed at diagnosis				Immunosuppressive	Inhibitor	Outcome
				PT-INR	APTT (sec)	FVIII:C (%)	FVIII inhibitor (BU)	therapy	recurrence	
Case 1	82	Female	Bullae with bleeding, Nasal hemorrhage	1.09	80	0.7	5.4	PSL^a^	No	Alive at 32 M
Case 2	56	Male	Left shoulder joint pain, Difficulty walking	0.96	68	1.3	3.2	PSL^a^, CPA^b^, Rituximab	No	Alive at 31 M
Case 3	58	Female	Intramuscular hematoma in left leg, Difficulty walking	0.97	94	1.1	5.9	PSL^a^	No	Alive at 18 M

The reference ranges for the PT-INR, APTT, and FVIII:C are 0.8-1.2, 25-35 sec, and 60-140%, respectively

The lower limit of detection of the Bethesda assay was 0.1 BU.

*TEG* Thromboelastography. *PT-INR* Prothrombin time-international normalized ratio; *APTT* Activated partial thromboplastin time

*FVIII:C* Factor VIII coagulant activity, *FVIII* Factor VIII, *BU* Bethesda units, *M* Months

^a^*PSL* Prednisolone, ^b^*CPA* Cyclophosphamide

### Case 2

A 56-year-old male with left shoulder joint pain and difficulty walking attended our clinic. He had had hyperuricemia and hyperlipidemia for 10 years and hypertension for 5 years before his visit. He saw an orthopedic surgeon for left shoulder pain. Left shoulder arthrocentesis revealed an intraarticular hemorrhage. 4 days later, he suffered from hip pain, and CT showed a hematoma in his iliopsoas muscle. He visited our clinic so that the reason for his bleeding tendency could be investigated. Laboratory tests showed a normal prothrombin time (PT), a prolonged APTT (68 sec), decreased FVIII:C (1.3%), and an increased FVIII inhibitor concentration (3.2 BU/mL) (Table 1). He was diagnosed with AHA. rFVIIa (90 μg/kg) was administered to control the progression of the pelvic hematoma. At that time, his R-value was prolonged (23.8 min). Immunosuppressive prednisolone therapy was also started. 4 days after the start of the rFVIIa treatment, the left shoulder pain had been ameliorated, and the patient’s R-value had decreased (Fig. 1B). The rFVIIa dosing interval started to be increased. However, after the rFVIIa dosing interval reached 12 hours, TEG-based analysis showed that the patient’s R-value had increased (14.7 min). At that time, his left shoulder pain recurred. Based on the prolonged R-value revealed by TEG, the rFVIIa dosing interval was reduced from every 12 hours to every 6 hours. In addition to the increased R-value, the patient’s FVIII:C level remained low at 1.7%, and cyclophosphamide (100 mg/day) was added as an immunosuppressive therapy. 1 month after the diagnosis, rFVIIa (dosing interval: every 8 hours) was discontinued based on an R-value of 8.2 min being demonstrated by TEG analysis just before the administration of the drug. At that time, his left shoulder pain had improved, and his iliopsoas hematoma had shrunk. The FVIII inhibitor was still detectable (1.9 BU/mL), and 375 mg/m^2^ of rituximab was administered twice, 1 week apart. After the second infusion of rituximab, the FVIII inhibitor became undetectable. After more than 2.5 years, the AHA has not relapsed.

### Case 3

A 58-year-old female, with a history of transient ischemic attacks (TIA) 2 years before her visit, suffered from leg pain and difficulty walking due to an intramuscular hematoma in her lower left leg. She had been taking cilostazol since the TIA. Laboratory tests revealed a normal PT, a prolonged APTT (94 sec), decreased FVIII:C (1.1%), and an increased FVIII inhibitor concentration (5.9 BU/mL) (Table 1). She was diagnosed with AHA and was treated with rFVIIa (90 μg/kg) to reduce the size of the hematoma in her left leg. The administration of 1 mg/kg prednisolone was also started. 7 days after admission, TEG performed just before the administration of rFVIIa revealed that the patient’s R-value was higher than the reference range (12.8 min) (Fig. 1C). At that time, although her leg pain had been relieved, new subcutaneous hemorrhages appeared. Based on the results of TEG, the dosing interval was not extended beyond 4 hours. 11 days after admission, the bleeding symptoms had been ameliorated. The patient’s R-value improved, and the rFVIIa dosing interval was increased to 6 hours. After the R-value reached 8.7 min (dosing interval: every 12 hours), rFVIIa was discontinued. 1 week later, the FVIII inhibitor became undetectable. She was able to return to daily life quickly after being discharged. After more than 1.5 years, the AHA has not relapsed.

## Discussion and conclusions

rFVIIa is used as a first-line bypassing agent during the treatment of AHA and has proven effective against clinically significant bleeding. However, it has a short half-life. The optimal rFVIIa dose and administration interval for use at the start of AHA treatment have already been clarified. Once hemostasis is achieved, the dosing interval of hemostatic agents should be increased [4]. However, as there are no established laboratory tests for determining the optimal rFVIIa dosing interval for controlling bleeding, the risk of thrombotic complications from overdosing or rebleeding from underdosing remains. This study described three cases in which the rFVIIa dosing interval was adjusted based on the results of TEG. Our cases suggest that performing TEG just before the administration of the next dose of rFVIIa would aid decisions regarding whether it is possible to extend the dosing interval of rFVIIa without causing bleeding complications. TEG produces various parameters. Our cases indicate that the R-value may be useful for assessing coagulation function prior to adjusting the dosing interval of rFVIIa after the administration of the initial dose of the drug.

TEG measures global clotting function [13]. In AHA, coagulation function often does not correlate with the levels of FVIII:C or FVIII inhibitor [2, 14]. Performing TEG instead of coagulation tests, such as the APTT, has been reported to be useful for assessing bleeding risk [15]. Conducting TEG-based analysis after the administration of rFVIIa was also demonstrated to be valuable for assessing the efficacy of rFVIIa in the presence of a neutralizing inhibitor of FVIII [10, 11]. However, there are no reports about increasing the rFVIIa dosing interval based on the results of TEG in AHA. Just before the next dose of rFVIIa is administered, the effect of the previous dose disappears. Hence, we considered that performing TEG-based analysis just before the administration of the next dose of rFVIIa may facilitate safe dosing interval increases during hemostatic therapy. In the cases described in the present study, the attending physicians decided that the rFVIIa dosing interval could be extended safely without increasing the risk of rebleeding if the R-value according to TEG was within the reference range (4.6-9.1 min). Our cases suggest that conducting TEG just before the administration of the next dose of rFVIIa helps guide increases in the rFVIIa dosing interval without increasing the risk of rebleeding.

TEG-based coagulation monitoring was conducted at the attending physician’s discretion in this study. Evaluations of coagulation function, especially those relating to the timing of changes in the rFVIIa dosing interval, were performed using TEG. Further studies based on regular prospective TEG-based monitoring are warranted to establish how TEG can be used to guide changes in the dosing interval and the discontinuance of rFVIIa. Clot strength also needs to be examined at different timepoints during rFVIIa treatment.

TEG-based analysis produces various parameters, including the reaction time (R time), kinetic time (K time), α angle, and the maximum amplitude. Although there is no established method for assessing coagulation function using TEG in AHA, both the R time and K time were reported to be useful for TEG-based monitoring of the effects of rFVIIa treatment [10, 11]. The R-value reflects the adequacy of coagulation factors [16]. Therefore, we assessed the R-value using TEG. In our cases, the levels of FVIII:C and FVIII inhibitor did not change during the early stages of immunosuppressive treatment for AHA (Fig. 1). On the other hand, the R-value according to TEG fluctuated to reflect coagulation function, and hence, it helped guide adjustments to the rFVIIa dosing interval. The patients’ clinical courses suggest that evaluating the R-value using TEG is a suitable way of assessing coagulation function when considering adjustments to the rFVIIa dosing interval. The R-value reference range reported by the manufacturer of the TEG system was used as a threshold for making decisions in our cases, and the attending physicians decided that extended the rFVIIa dosing interval would increase the risk of rebleeding if the R-value was higher than the reference range. Additional investigations are needed to examine the optimal TEG-derived R-value threshold for determining appropriate rFVIIa dosing intervals.

rFVIIa is an expensive drug. Although it is crucial to administer enough rFVIIa to control bleeding and prevent rebleeding, the amount of rFVIIa used affects medical costs. At our institution, TEG-based analysis of coagulation function was conducted in 3 of 6 consecutive patients who received rFVIIa treatment for AHA between October 2016 and September 2020. The total dose and number of doses of rFVIIa were compared between the patients that did (TEG+ group) and did not (TEG- group) undergo TEG-based coagulation function monitoring. One patient in the TEG- group suffered from rebleeding in the iliopsoas muscle and received additional rFVIIa therapy. He died 9 months after the diagnosis of AHA due to a decreased ability to perform ADL. None of the other patients experienced rebleeding or death. The mean total dose of rFVIIa was 10.5 mg/kg and 17.6 mg/kg in the TEG+ and TEG- groups, respectively. The mean number of doses of rFVIIa administered to the patients in the TEG+ and TEG- groups were 111.7 and 171.0, respectively. Although the number of analyzed cases was too small for statistical analyses to be performed, smaller rFVIIa dosages result in lower medical costs. More prospective studies are needed to clarify whether TEG-based monitoring reduces the total rFVIIa dose required.

Our hospital is located in a small city in Japan and does not specialize in hemophilia. The majority of patients admitted to our hospital are elderly. Bleeding events can quickly impair the ability of elderly patients to perform ADL, and attending physicians may use rFVIIa more abundantly in the elderly than in younger patients. It is important to establish a way to safely reduce the dosing frequency of rFVIIa, which can reduce medical costs. Due to the rarity of AHA, it is recommended that patients with AHA are treated at hemophilia care centers. Further prospective evaluations are needed to elucidate the association between TEG-based monitoring and the total rFVIIa dosage among patients treated at specialized centers.

In conclusion, rFVIIa for AHA may be tapered and discontinued safely without increasing the risk of rebleeding by performing TEG just before the administration of rFVIIa. It is important to establish a way of adjusting the rFVIIa dosing interval during treatment. Further studies are warranted to determine the optimal way of using TEG to increase the rFVIIa dosing interval.

## Data Availability

The datasets analyzed during the current study are not publicly available due to the need to protect patient privacy, but are available from the corresponding author on reasonable request.

## Abbreviations

AHA: Acquired hemophilia A
rFVIIa: Recombinant activated factor VII
TEG: Thromboelastography
R: Reaction rate time
ADL: Activities of daily living
APTT: Activated partial thromboplastin time
FVIII:C: Factor VIII coagulant activity
FVIII: Factor VIII
BU: Bethesda units
CT: Computed tomography
ROTEM: Rotational thromboelastometry
PT: Prothrombin time
